# Tranexamic Acid for Acute Spontaneous Intracerebral Hemorrhage: A Meta-Analysis of Randomized Controlled Trials

**DOI:** 10.3389/fneur.2021.761185

**Published:** 2021-12-20

**Authors:** Yu Guo, Xin-Mei Guo, Rui-Li Li, Kai Zhao, Qiang-Ji Bao, Jin-Cai Yang, Qiang Zhang, Ming-Fei Yang

**Affiliations:** ^1^Graduate School, Qinghai University, Xining, China; ^2^Biomedical Engineering Research Center, Kunming Medical University, Kunming, China; ^3^Neurological Intensive Care Department, Shengli Oilfield Central Hospital, Dongying, China; ^4^Department of Neurosurgery, Qinghai Provincial People's Hospital, Xining, China

**Keywords:** cerebral hemorrhage, hematoma, tranexamic acid, randomized controlled trial, meta-analysis

## Abstract

**Background:** The role of tranexamic acid (TXA) in preventing hematoma expansion (HE) in patients with acute spontaneous intracerebral hemorrhage (ICH) remains unclear. We aim to investigate the efficacy and safety of TXA in acute spontaneous ICH with a particular focus on subgroups.

**Methods:** Randomized controlled trials (RCTs) were retrieved from CENTRAL, Clinicaltrials.gov, EMBASE, PubMed, and WHO ICTRP. The primary outcome measurement was HE. The secondary outcome measurements included 3-month poor functional outcome (PFO), 3-month mortality, and major thromboembolic events (MTE). We conducted subgroup analysis according to the CT markers of HE (standard-risk population and high-risk population) and the time from onset to randomization (>4.5 and ≤4.5 h).

**Results:** We identified seven studies (representing five RCTs) involving 2,650 participants. Compared with placebo, TXA may reduce HE on subsequent imaging (odd ratio [OR] 0.825; 95% confidence interval [CI] 0.692–0.984; *p* = 0.033; I^2^ = 0%; GRADE: moderate certainty). TXA and placebo arms did not differ in the rates of 3-month PFO, 3-month mortality, and MTE. Subgroup analysis indicated that TXA reduced the risk of HE in the high-risk population with CT markers of HE (OR 0.646; 95% CI 0.503–0.829; *p* = 0.001; I^2^ = 0 %) and in patients who were treated within 4.5 h of symptom onset (OR 0.823; 95% CI 0.690–0.980; *p* = 0.029; I^2^ = 0%), but this protective effect was not observed in the standard-risk population and patients who were treated over 4.5 h of symptom onset.

**Conclusions:** Tranexamic acid (TXA) may decrease the risk of HE in patients with acute spontaneous ICH. Importantly, the decreased risk was observed in patients who were treatable within 4.5 h and with a high risk of HE, but not in those who were treatable over 4.5 h and in standard-risk population. However, PFO or mortality at 3 months did not significantly differ between patients who received TXA and those who received placebo. TXA is safe for acute spontaneous ICH without increasing MTE.

## Introduction

Spontaneous intracerebral hemorrhage (ICH) is one of the leading causes of disability and death worldwide, and is one of the serious global public health and socioeconomic burdens. Globally, up to 3 million people die from ICH each year, accounting for 5% of all human deaths, while an estimated 18 million people suffer from the sequelae of ICH (1, 2). Although an organized in-patient (stroke unit) care has been shown to contribute in reducing disability and mortality, there is no strong evidence-based acute therapy that is specific to acute spontaneous ICH (3, 4).

Age, neurological deficit, hemorrhage cause, location, and hematoma volume are the main determinants of clinical outcomes in patients with ICH (5, 6). Among them, hematoma volume is the most important factor. Roughly one-third of acute spontaneous ICH are complicated by hematoma expansion (HE), which most often occurs within the first few hours, but could also occur at up to 24 h, presenting a target window for intervention (7, 8). Emergency medical services are traditionally established to bring in patients with acute stroke with haste for diagnosis and treatment within 4.5 h of symptom onset. In addition, tranexamic acid (TXA) therapy is theoretically more suitable for patients with high-risk for ICH growth, such as patients with early computed tomography (CT) markers of HE. CT markers, including black hole sign, blend sign, island sign, and spot sign, have been used as reliable predictors for early HE in patients with acute spontaneous ICH (9–14). Therefore, it seems feasible to identify patients at high risk for HE early, and then for early therapeutic intervention to improve the clinical outcomes of patients with acute spontaneous ICH.

The researchers are looking for a safe acute therapy to reduce the risk of HE and improve the outcome for acute spontaneous ICH patients. TXA is an antifibrinolytic agent that reduces bleeding by inhibiting plasminogen activation and fibrinolysis. It has been shown that it can reduce perioperative blood loss and risk of blood transfusion (15, 16). In addition, trauma guidelines have recommended the early use of TXA in patients with traumatic ICH, as TXA can provide a survival benefit without increasing its adverse events (17, 18). However, the role of TXA in preventing HE and in improving outcome in patients with acute spontaneous ICH remains unclear.

Several randomized controlled trials (RCTs) evaluating the efficacy and safety of TXA in patients with acute spontaneous ICH have been performed in recent years. However, the existing RCTs and previously published systematic reviews have reported fragmentary and conflicting results. Some studies have shown a protective association between TXA use and ICH growth. Other studies have found no such relationship. These studies differed in their study populations. Herein, we performed meta-analysis on the available RCTs to determine the following: (1) the effectiveness and safety of TXA administration, compared against placebo or open control, in adults with acute spontaneous ICH; (2) how this differs between the standard-risk population and the high-risk population (those with ICH with CT markers of HE); and (3) how this differs between the range within 4.5 h and over 4.5 h of the time from onset to randomization.

## Methods

### Guidance and Protocol

This systematic review and meta-analysis were performed in compliance with the Cochrane Handbook for Systematic Reviews of Interventions (19) and was reported in accordance with the Preferred Reporting Items for Systematic Reviews and Meta-Analyses (PRISMA) guidelines (20). The review protocol was prospectively registered in International Platform of Registered Systematic Review and Meta-analysis Protocols (INPLASY) under number INPLASY202170032. The PRISMA 2020 checklist is available in the Supplementary Table 1.

### Eligibility Criteria

We considered studies to be eligible if they met the following criteria: (i) Types of studies: RCT published in peer-reviewed medical journals; (ii) Types of participants: Adult patients (aged ≥18) with spontaneous ICH and are treatable within 24 h of symptom onset; (iii) Types of interventions: TXA at any dose versus placebo; (iv) Types of outcome measures: The outcome measurements were HE (defined as hematoma growth >33% and/or >6 ml), 3-month poor functional outcome (PFO; defined as modified Rankin Scale score 4–6), 3-month mortality, and major thromboembolic events (MTE; according to the definition provided in each study).

### Search Strategy

We conducted a comprehensive literature search on Cochrane Central Register of Controlled Trials (CENTRAL), ClinicalTrials.gov, Embase, PubMed, and the World Health Organization International Clinical Trials Registry Platform (WHO ICTRP) from inception until July 3, 2021 with no language restriction. RCTs investigating the effects of TXA in adult patients with acute spontaneous ICH were included. Controlled vocabulary (i.e., MeSH and Emtree) and keywords were used. Search terms included TXA, ICH, RCT, and their variants. The complete search strategy is available in the Supplementary Table 2. We manually screened the reference lists of eligible trials and previous relevant reviews for additional studies.

### Study Selection

After records were imported into the Zotero reference management software (www.zotero.org), duplicate records were manually removed. Two reviewers independently screened the titles and abstracts for relevance, and labeled records as probably included and excluded in duplicates. If records are deemed potentially relevant by either reviewer, the full-text articles were retrieved to assess its eligibility. Disagreements were resolved by discussion, and by a third-party adjudication if required.

### Data Extraction

Two reviewers independently extracted data and in duplicate using a standardized form. We extracted the following information from included trials: (i) trial characteristics: study title, fist author name, year of publication, country of origin, study design, and number of participants; (ii) patient characteristics: CT markers of HE, age, sex, and baseline National Institutes of Health Stroke Scale (NIHSS) score; (iii) intervention characteristics: treatment administration time and TXA dose; and (iv) data on outcomes of interest, etc.

### Risk of Bias Assessment

Two reviewers have assessed the risk of bias of each trial independently and in duplicates using the Cochrane Collaboration's tool based on the recommendations of the Cochrane Handbook for Systematic Reviews of Intervention (21). The following items for risk of bias were examined: random sequence generation (selection bias), allocation concealment (selection bias), blinding of participants and personnel (performance bias), blinding of outcome assessment (detection bias), incomplete data outcome (attrition bias), selective reporting (reporting bias), and, other biases (such as stopping early and funding source). The risk of bias was determined as “high risk,” “unclear risk,” or “low risk.” Disagreements were resolved by discussion, and third-party adjudication if required.

### Quality of Evidence

Two reviewers assessed the overall certainty of evidence for each outcome using the Grading Recommendations Assessment, Development and Evaluation (GRADE) system (22). The items assessed included risk of bias, inconsistency, indirectness, imprecision, and publication bias. The evidence quality was classified as very low, low, moderate, and high. Disagreements were resolved by discussion, and third-party adjudication if required.

### Statistical Analysis

We calculated odds ratios (ORs) and their corresponding 95% CIs to measure the effect size while comparing TXA vs. control among patients with acute spontaneous ICH. Meta-analyses were performed using DerSimonian and also Laird random-effects models accounting for clinical heterogeneity (23). A *p*-value of < 0.05 was considered statistically significant. The heterogeneity between trials was assessed using the Cochran *Q* test (with *p* < 0.1 indicating significance) and was quantified by the I^2^ statistic (for which a value of 50% or greater was considered to represent significant heterogeneity) (24). Publication bias across individual trials was graphically evaluated using a funnel plot and also with the Egger's test at a significance level of *p* < 0.05 (25). We performed one *post hoc* sensitivity analysis, restricting to trials with a low risk of bias, to test the robustness of our findings. We also conducted subgroup analysis according to different subjects (high-risk population or standard-risk population) and the time from onset to randomization ( ≤ 4.5 or >4.5 h). High-risk population was defined as patients with CT markers of HE in patients with ICH. Standard-risk population was defined as general ICH population, without further screening of CT markers of HE. Specific data of the intervention and control groups were extracted from publications. If the original data were not available, the adjusted ORs, as the indicators of the outcomes, were alternatively extracted. All statistical analyses were conducted with Comprehensive Meta-Analysis software (version 3) and Stata software (version 15).

## Results

### Result of Literature Search

**Figure 1:**
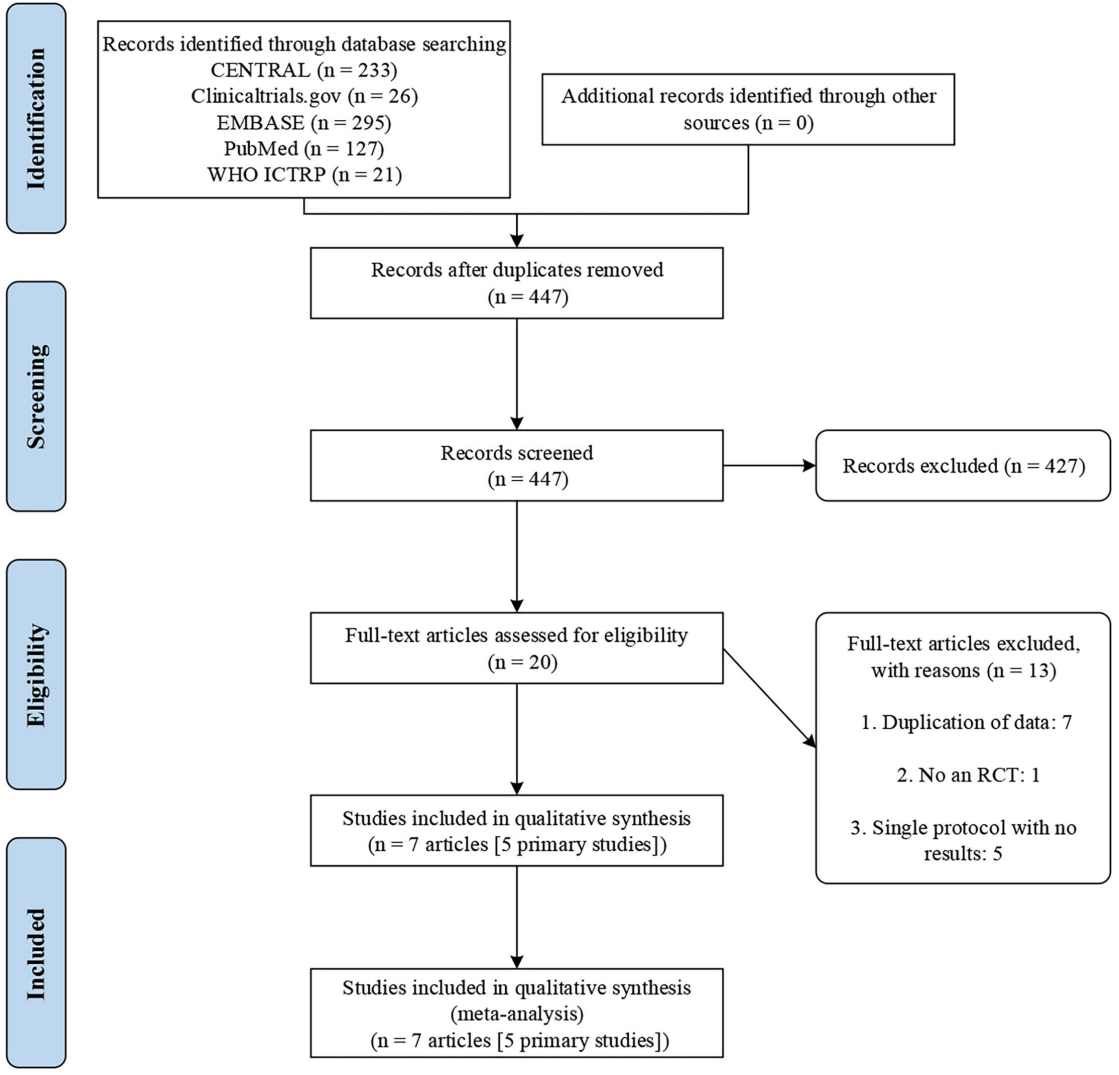
PRISMA flowchart.

The initial search yielded 702 records. We excluded 275 duplicates and a further 427 records after title and abstract screening. Then, 20 potentially eligible articles were retained for full-text evaluation. Finally, seven trial reports (26–32) representing 5 RCTs were included according to the inclusion criteria. Figure 1 illustrates the study selection process.

### Characteristics of Eligible Studies

**Figure 2:**
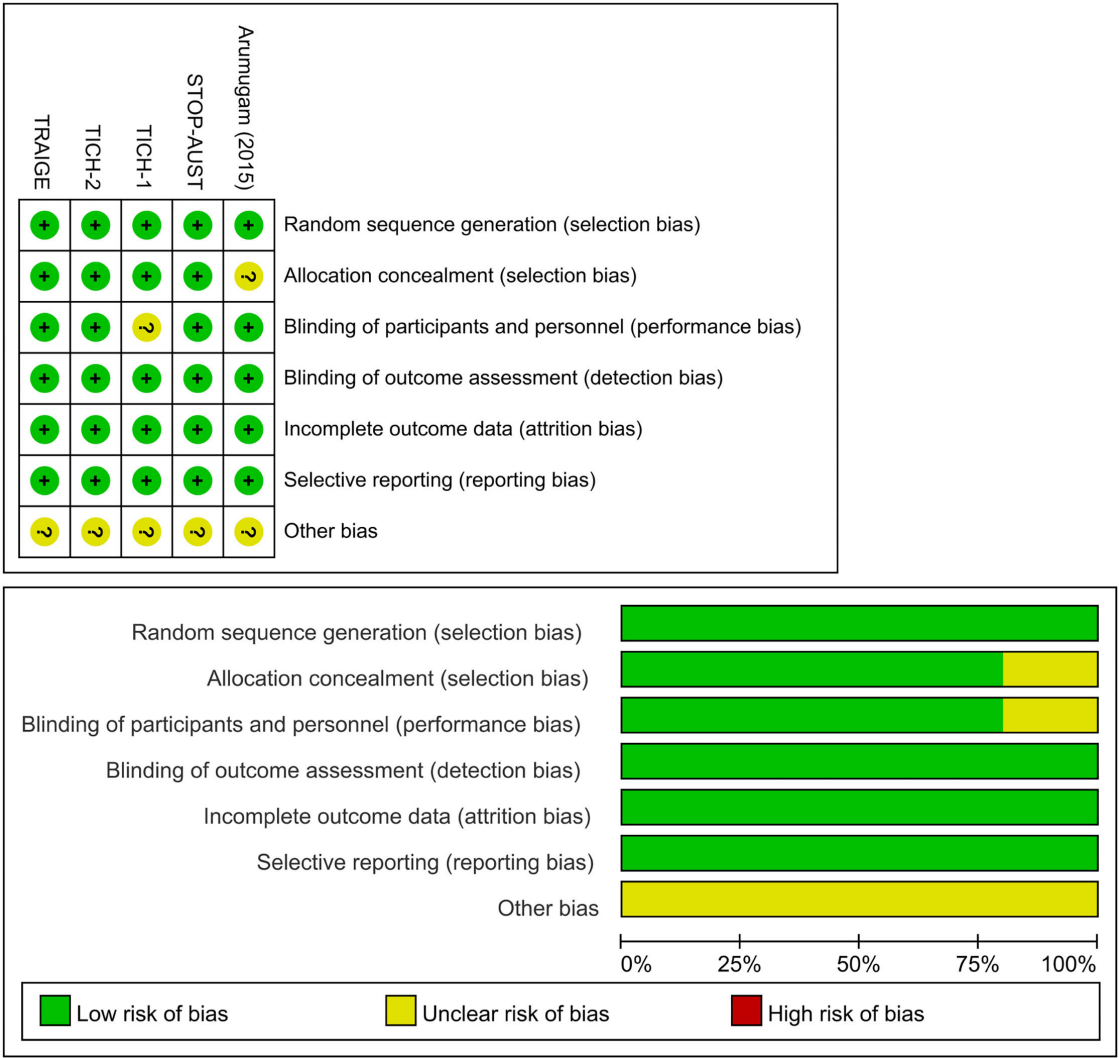
Risk of bias assessment.

Baseline characteristics of the included seven trial reports are summarized in Table 1. Of the two additional trial reports, one was a *post hoc* analysis (31), and the other was a pre-specified subgroup analysis (32) of a previously published trial (30). The 7 trial reports were published from 2014 to 2021, with sample sizes ranging from 24 to 2,325 subjects, and a total of 2,650 subjects. Three trials were multicenter studies (27, 28, 30), while two were conducted at a single center (26, 29). The subjects were mainly men and the mean age of subjects ranged from 53 to 73 years. The baseline NIHSS scores ranged from 12 to 18. Two trials (27, 28) enrolled acute spontaneous ICH patients susceptible to HE based on imaging assessment; however, the majority of patients included were general acute spontaneous ICH patients. The TXA administration time was varied among studies: within 4.5 h in one trial (28), within 8 h in three trials (26, 27, 30), and, up to 24 h in one trial (29). The dosage of TXA was consistent across included trials with the most common regimen being a loading dose of 1 g, followed by a maintenance dose of 1 g over 8 h. Overall, three trials (27, 28, 30) were categorized as being at low risk of bias and 2 (26, 29), as being unclear. Details of the risk of bias are presented in Figure 2 and Supplementary Table 3.

**Table 1:** Baseline characteristics of included studies.

**References**	**Study title**	**Country**	**Study design**	**Total-n**	**Population**	**CT signs**	**Age^†^-y**	**Male-%**	**Baseline NIHSS^†^**	**Treatmentadmission time**	**TXA dose**	**Follow up-d**
Arumugam et al. (26)	–	Malaysia	sRCT	30	Standard-risk population	–	53.9	60.0	NR	Within 8 h	1 g TXA bolus followed by 1 g TXA maintenance	1
Liu et al. (27)	TRAIGE	China	mRCT	171	High-risk population	Spot sign, black hole sign, and blend sign	55.9	72.5	11.0	Within 8 h	1 g TXA bolus followed by 1 g TXA maintenance	90
Meretoja et al. (28)	STOP-AUST	Australia	mRCT	100	High-risk population	Spot sign	73.0/71.0^*^	62.0	14.0/12.0^*^	Within 4.5 h	1 g TXA bolus followed by 1 g TXA maintenance	90
Sprigg et al. (29)	TICH-1	England	sRCT	24	Standard-risk population	-	68.1	62.5	15.1	Within 24 h	1 g TXA bolus followed by 1 g TXA maintenance	90
Sprigg et al. (30)	TICH-2	Multinational	mRCT	2325	Standard-risk population	-	68.9	56.0	13.0/13.0^*^	Within 8 h	1 g TXA bolus followed by 1 g TXA maintenance	90
Law et al. (31)	*Post-hoc* analysis of TICH-2	Multinational	mRCT	2077	High-risk population	Black hole sign, blend sign, and island sign	NR	NR	NR	Within 8 h	1 g TXA bolus followed by 1 g TXA maintenance	90
Ovesen et al. (32)	Pre-specified subgroup of TICH-2	Multinational	mRCT	254	High-risk population	Spot sign	64.7	57.8	18.0/16.5^*^	Within 8 h	1 g TXA bolus followed by 1 g TXA maintenance	90

*The values are given as the mean*.

* *The values are given as the median of TXA/placebo*.

*mRCT, multisite randomized controlled trial; NIHSS, National Institutes of Health Stroke Scale; NR, not report; sRCT, single site randomized controlled trial; TXA, tranexamic acid*.

### Association Between TXA and Outcomes

Table 2 summarizes the overview of the association between TXA and various clinical outcomes. GRADE summary of findings is available in the Table 2 and Supplementary Table 4.

**Table 2:** Overview of the safety and efficacy analyses on different endpoints.

**Outcome**	**Trials, n**	**Result**			**Certainty**	**Importance**
		**OR (95% CI)**	***p* Value**	**Heterogeneity (I^**2**^, *p* for Cochran Q)**		
HE	5	0.825 (0.692–0.984)	0.033	I^2^ = 0.000%, *p =* 0.732	Moderate ⊕⊕⊕⊕○	Critical
PFO (3 mo)	4	0.991 (0.849–1.158)	0.914	I^2^ = 0.000%, *p =* 0.871	High ⊕⊕⊕⊕⊕	Critical
Mortality (3 mo)	4	1.020 (0.843–1.234)	0.834	I^2^ = 0.000%, *p =* 0.623	High ⊕⊕⊕⊕⊕	Critical
MTE	4	1.092 (0.721–1.655)	0.678	I^2^ = 0.000%, *p =* 0.848	Moderate ⊕⊕⊕⊕○	Critical

*CI, confidence interval; HE, hematoma expansion; MTE, major thromboembolic events; OR, odd ratio; PFO, poor functional outcome*.

### Primary Outcome Measurement

**Figure 3:**
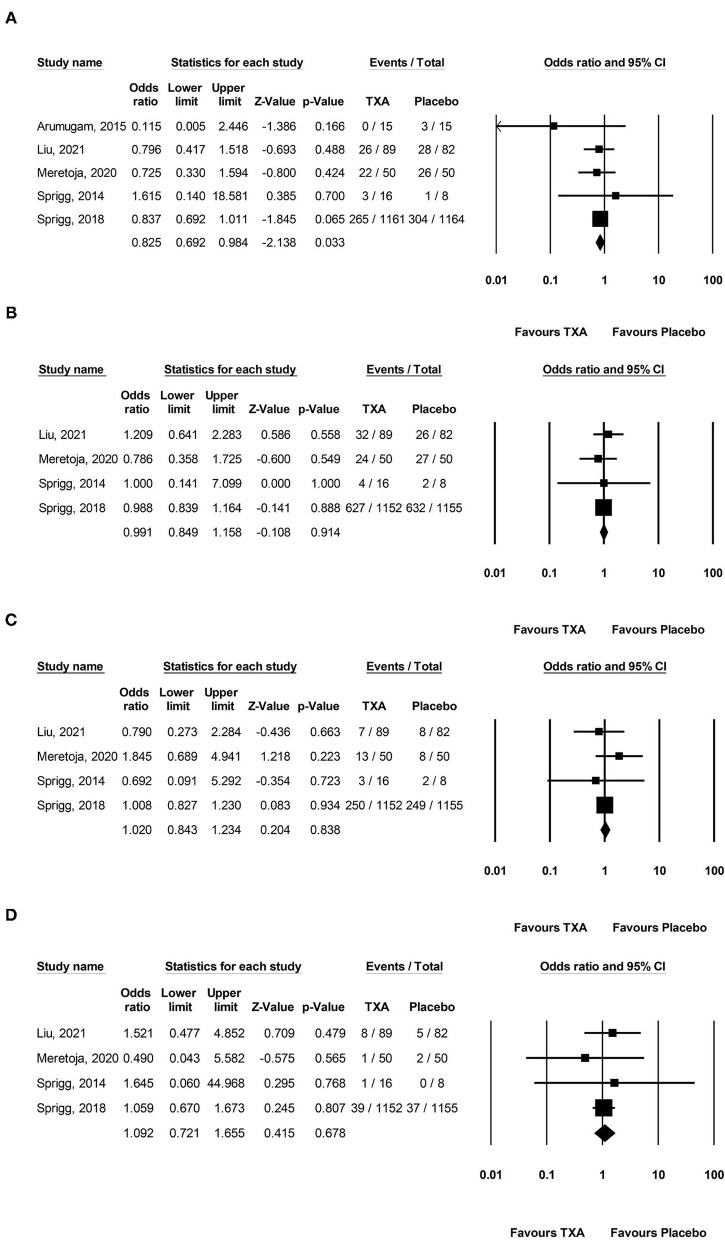
Forest plot comparing the risk of **(A)** hematoma expansion, **(B)** 3-month poor functional outcome, **(C)** 3-month mortality, and **(D)** major thromboembolic events between the TXA and placebo groups. TXA, tranexamic acid.

**Figure 4:**
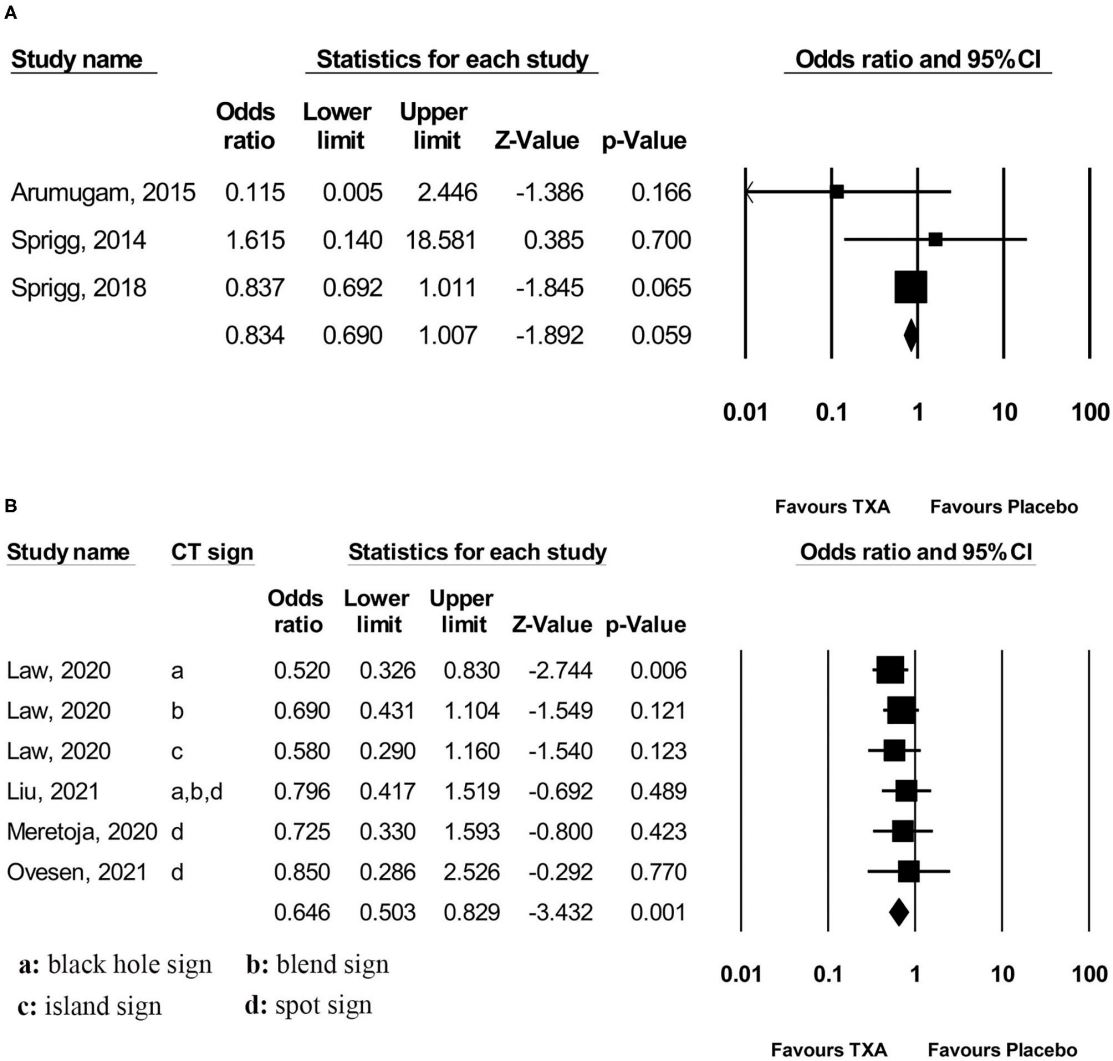
Subgroup analysis of primary outcome measurement: **(A)** standard-risk population and **(B)** high-risk population. TXA, tranexamic acid.

**Figure 5:**
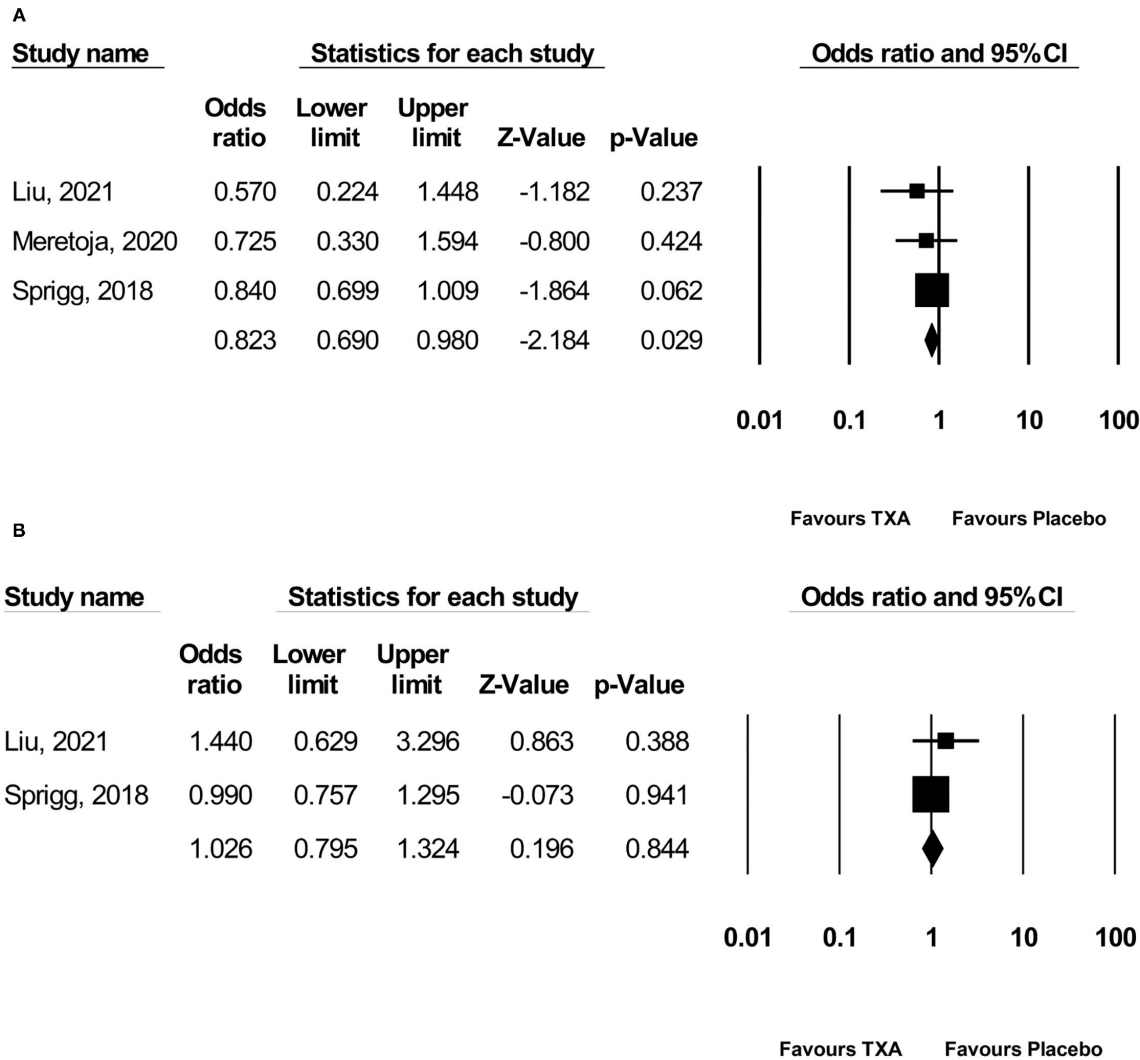
Subgroup analysis of primary outcome measurement: **(A)** ≤ 4.5 h and **(B)** > 4.5 h. TXA, tranexamic acid.

Pooled analysis found that TXA administration may reduce HE on subsequent neuroimaging (OR 0.825; 95 % CI 0.692–0.984; *p* = 0.033; I^2^ = 0 % and chi-square *p* = 0.732; Figure 3A). The GRADE certainty of the evidence was moderate. Sensitivity analysis showed similar results when limited to trials with low risk of bias (OR 0.828; 95% CI 0.693–0.988; *p* = 0.036; I^2^ = 0 % and chi-square *p* = 0.935; Supplementary Appendix 1). Subgroup analyses indicated that TXA reduced the risk of HE in the high-risk population (OR 0.646; 95% CI 0.503–0.829; *p* = 0.001; I^2^ = 0 % and chi-square *p* = 0.886; Figure 4B) and patients who were treatable within 4.5 h of symptom onset (OR 0.823; 95% CI 0.690–0.980; *p* = 0.029; I^2^ = 0% and chi-square *p* = 0.690; Figure 5A), but not in the standard-risk population (OR 0.834; 95% CI 0.690–1.007; *p* = 0.059; I^2^ = 0 % and chi-square *p* = 0.388; Figure 4A), and patients who were treatable over 4.5 h of symptom onset (OR 1.026; 95% CI 0.795–1.324; *p* = 0.844; I^2^ = 0 % and chi-square *p* = 0.399; Figure 5B).

### Secondary Outcome Measurements

However, TXA probably had no effect on a 3-month PFO (OR 0.991; 95 % CI 0.849–1.158; *p* = 0.914; I^2^ = 0 % and chi-square *p* = 0.871; GRADE: high certainty; Figure 3B), 3-month mortality (OR 1.020; 95 % CI 0.843–1.234; *p* = 0.834; I^2^ = 0 % and chi-square *p* = 0.623; GRADE: high certainty; Figure 3C), and MTE (OR 1.092; 95% CI 0.721–1.655; *p* = 0.678; I^2^ = 0 % and chi-square *p* = 0.848; GRADE: high certainty; Figure 3D). To confirm the robustness of our findings, we conducted sensitivity analyses using data from trials categorized as low risk of bias for various clinical outcomes. Sensitivity analysis for the rate of each endpoint showed that the overall effect of TXA therapy was consistent with the overall estimates from all studies (Supplementary Appendix 1). There was no significant difference in the 3-month PFO, 3-month mortality, and MTE in the high-risk population vs. standard-risk population subgroups (Supplementary Appendix 2).

### Publication Bias

For the safety and efficacy analyses on different endpoints, funnel plot and the Egger's test revealed no evidence of asymmetry (Supplementary Figure 1). However, the results from such analyses should be treated with considerable caution based on a limited number of trials.

## Discussion

### Principal Findings

Our meta-analysis has comprehensively and systematically reviewed the current available literature that compared TXA with placebo for treating acute spontaneous ICH, and we obtained three major findings. Firstly, in patients with spontaneous ICH, TXA may decrease HE on subsequent imaging. However, TXA probably had no effect on PFO and on mortality at 3 months. The use of TXA was safe without increasing thromboembolic complications. Secondly, further analyses restricted to patients with spontaneous ICH susceptible to HE based on imaging assessment have showed consistent results, but this protective effect was not found in the standard-risk population. CT markers of HE may be a useful determinant of benefit from TXA administration in acute spontaneous ICH patients. Thirdly, early treatment (within 4.5 h after stroke onset) seemed to show more benefits in preventing HE, but this protective effect was not observed in the patients who were treatable over 4.5 h.

### Relation to Other Systematic Reviews

Two previous systematic reviews on the similar topic have been published (33, 34). One of them evaluated the treatment of acute spontaneous ICH with different hemostatic therapies (including TXA) and the results showed that there was no evidence of either benefit or harm from TXA for people with ICH (33). The other one evaluated the effect of TXA on acute spontaneous ICH and found that TXA could reduce HE in ICH (34). In line with the second study (34), our meta-analysis also found that TXA may decrease HE on subsequent imaging. Besides, we found that the decreased risk was observed in patients who were treatable within 4.5 h and in high-risk population, but not in those who were treatable over 4.5 h and in standard-risk population. In summary, our meta-analysis further confirms that TXA is an effective and safe drug for the treatment of acute spontaneous ICH and that CT markers of HE, as well as early time windows, are useful clinical determinants of TXA administration in acute spontaneous ICH patients. However, some differences also should be noted. First, previous meta-analyses included <4 RCTs. Our meta-analysis identified another three recent studies and further reinforced earlier results of previous meta-analyses. Second, we pooled RCTs data with a random-effects model accounting for clinical heterogeneity to ensure a more conservative estimate of the efficacy and safety of TXA for the treatment of acute spontaneous ICH. Third, we evaluated the certainty of evidence using GRADE approach to facilitate clinical decision-making.

### Strengths and Weaknesses of the Review

This systematic review and meta-analysis have several strengths including a pre-registered protocol, a comprehensive literature search, a duplicate and independent screening and data extraction, and GRADE assessment of certainty of evidence. However, certain limitations of this meta-analysis need to be acknowledged. First, subgroup analysis for each CT markers of HE was not performed because of the lack of data. Thus, the difference in efficacy for patients with different CT markers of HE was not determined. Second, the inclusion of the *post hoc* RCT would lead to a declined quality of subgroup analysis that we conducted.

### Future Perspectives

The study limitations mentioned above provided several considerations for future studies. In particular, the difference in efficacy for patients with different CT markers of HE should be determined. Subgroup analyses according to time from onset to randomization have found that early treatment (within 4.5 h) seemed to show more benefit. The proposed time window could also be used for hemostatic therapy in future research.

## Conclusions

In the overall population with acute spontaneous ICH, TXA may decrease the risk of HE on subsequent imaging. Patients with high-risk of HE predicted by CT markers and patients who are treatable within 4.5 h may both have the greatest risk reduction of HE. However, TXA probably has no effect on PFO or mortality at 3 months. The use of TXA probably does not increase the risk of MTE.

## Data Availability Statement

The original contributions presented in the study are included in the article/Supplementary Material, further inquiries can be directed to the corresponding author/s.

## Author Contributions

YG, X-MG, and R-LL: acquisition of data, analysis and interpretation of data, and drafting the article. KZ, Q-JB, J-CY, and QZ: critical revision of the manuscript for important intellectual content. M-FY: conception and design of the study and critical revision of the manuscript for important intellectual content. All authors have read and approved the final version of the manuscript.

## Funding

This study was supported by the Science and Technology Department of Qinghai Province (Nos. 2019-ZJ-7040 and 2020-ZJ-774). The funding had no role in the study design, data collection and analysis, decision to publish, or preparation of the manuscript.

## Conflict of Interest

The authors declare that the research was conducted in the absence of any commercial or financial relationships that could be construed as a potential conflict of interest.

## Publisher's Note

All claims expressed in this article are solely those of the authors and do not necessarily represent those of their affiliated organizations, or those of the publisher, the editors and the reviewers. Any product that may be evaluated in this article, or claim that may be made by its manufacturer, is not guaranteed or endorsed by the publisher.
