# Supplementary material for: Tranexamic Acid for Acute Spontaneous Intracerebral Hemorrhage: A Meta-Analysis of Randomized Controlled Trials
Source: Front Neurol. 2021 Dec 20;12:761185. doi: 10.3389/fneur.2021.761185 (PMC8720763; doi:10.3389/fneur.2021.761185)

Supplementary Materials

**Supplement to:** Yu Guo, Xin-Mei Guo, Rui-Li Li, Kai Zhao, Qiang-Ji Bao, Jin-Cai Yang, Qiang Zhang, Ming-Fei Yang. Tranexamic acid for acute spontaneous intracerebral hemorrhage: a meta-analysis with a particular focus on subgroups.

**Table of Contents**

**[Supplementary Table 1.](#_Toc49954750)** [PRISMA 2020 checklist](#_Toc49954750) 2-6

**[Supplementary Table 2.](#_Toc49954750)** [CENTRAL, Clinicaltrials.gov, EMBASE, PubMed, WHO ICTRP Search strategy](#_Toc49954750) 7-8

**[Supplementary Table 3.](#_Toc49954757)** [Assessment of risk of bias of individual trials](#_Toc49954757) 9-12

**[Supplementary Table 4.](#_Toc49954757)** [GRADE assessment on the certainty of evidence](#_Toc49954757) 13

**[Supplement Figure 1.](#_Toc49954758)** [Funnel plot and Egger's test.](#_Toc49954758) [(A) hematoma expansion, (B) 3-month poor functional outcome, (C) 3-month mortality, and (D) major thromboembolic events 1](#_Toc49954758)4-15

**[Supplementary Appendix 1.](#_Toc49954758)** [Post-hoc sensitivity analysis](#_Toc49954758) 16-17

**[Supplementary Appendix 2.](#_Toc49954758)** [Subgroup analysis was conducted according to CT sign 1](#_Toc49954758)8-20

**[Supplementary Table 1.](#_Toc49954750)** [PRISMA 2020 checklist](#_Toc49954750)

| **Section and Topic** | **Item #** | **Checklist item** | **Location where item is reported** |
| --- | --- | --- | --- |
| **TITLE** | | |  |
| Title | 1 | Identify the report as a systematic review. | Page 1 |
| **ABSTRACT** | | |  |
| Abstract | 2 | See the PRISMA 2020 for Abstracts checklist. | Page 2-3 |
| **INTRODUCTION** | | |  |
| Rationale | 3 | Describe the rationale for the review in the context of existing knowledge. | Page 3-4 |
| Objectives | 4 | Provide an explicit statement of the objective(s) or question(s) the review addresses. | Page 4 |
| **METHODS** | | |  |
| Eligibility criteria | 5 | Specify the inclusion and exclusion criteria for the review and how studies were grouped for the syntheses. | Page 5 |
| Information sources | 6 | Specify all databases, registers, websites, organisations, reference lists and other sources searched or consulted to identify studies. Specify the date when each source was last searched or consulted. | Page 5-6 |
| Search strategy | 7 | Present the full search strategies for all databases, registers and websites, including any filters and limits used. | Page 5-6 |
| Selection process | 8 | Specify the methods used to decide whether a study met the inclusion criteria of the review, including how many reviewers screened each record and each report retrieved, whether they worked independently, and if applicable, details of automation tools used in the process. | Page 6 |
| Data collection process | 9 | Specify the methods used to collect data from reports, including how many reviewers collected data from each report, whether they worked independently, any processes for obtaining or confirming data from study investigators, and if applicable, details of automation tools used in the process. | Page 6-7 |
| Data items | 10a | List and define all outcomes for which data were sought. Specify whether all results that were compatible with each outcome domain in each study were sought (e.g. for all measures, time points, analyses), and if not, the methods used to decide which results to collect. | Page 5 |
|  | 10b | List and define all other variables for which data were sought (e.g. participant and intervention characteristics, funding sources). Describe any assumptions made about any missing or unclear information. | Page 5 |
| Study risk of bias assessment | 11 | Specify the methods used to assess risk of bias in the included studies, including details of the tool(s) used, how many reviewers assessed each study and whether they worked independently, and if applicable, details of automation tools used in the process. | Page 7 |
| Effect measures | 12 | Specify for each outcome the effect measure(s) (e.g. risk ratio, mean difference) used in the synthesis or presentation of results. | Page 7-8 |
| Synthesis methods | 13a | Describe the processes used to decide which studies were eligible for each synthesis (e.g. tabulating the study intervention characteristics and comparing against the planned groups for each synthesis (item #5)). | Page 5 |
|  | 13b | Describe any methods required to prepare the data for presentation or synthesis, such as handling of missing summary statistics, or data conversions. | Page 8 |
|  | 13c | Describe any methods used to tabulate or visually display results of individual studies and syntheses. | Page 6 |
|  | 13d | Describe any methods used to synthesize results and provide a rationale for the choice(s). If meta-analysis was performed, describe the model(s), method(s) to identify the presence and extent of statistical heterogeneity, and software package(s) used. | Page 8 |
|  | 13e | Describe any methods used to explore possible causes of heterogeneity among study results (e.g. subgroup analysis, meta-regression). | Page 8 |
|  | 13f | Describe any sensitivity analyses conducted to assess robustness of the synthesized results. | Page 8 |
| Reporting bias assessment | 14 | Describe any methods used to assess risk of bias due to missing results in a synthesis (arising from reporting biases). | Page 7 |
| Certainty assessment | 15 | Describe any methods used to assess certainty (or confidence) in the body of evidence for an outcome. | Page 7 |
| **RESULTS** | | |  |
| Study selection | 16a | Describe the results of the search and selection process, from the number of records identified in the search to the number of studies included in the review, ideally using a flow diagram. | Page 8-9 |
|  | 16b | Cite studies that might appear to meet the inclusion criteria, but which were excluded, and explain why they were excluded. | None |
| Study characteristics | 17 | Cite each included study and present its characteristics. | Page 9 |
| Risk of bias in studies | 18 | Present assessments of risk of bias for each included study. | Page 9 |
| Results of individual studies | 19 | For all outcomes, present, for each study: (a) summary statistics for each group (where appropriate) and (b) an effect estimate and its precision (e.g. confidence/credible interval), ideally using structured tables or plots. | Page 10 |
| Results of syntheses | 20a | For each synthesis, briefly summarise the characteristics and risk of bias among contributing studies. | Page 10-11 |
|  | 20b | Present results of all statistical syntheses conducted. If meta-analysis was done, present for each the summary estimate and its precision (e.g. confidence/credible interval) and measures of statistical heterogeneity. If comparing groups, describe the direction of the effect. | Page 10-11 |
|  | 20c | Present results of all investigations of possible causes of heterogeneity among study results. | None |
|  | 20d | Present results of all sensitivity analyses conducted to assess the robustness of the synthesized results. | Page 10-11 |
| Reporting biases | 21 | Present assessments of risk of bias due to missing results (arising from reporting biases) for each synthesis assessed. | Page 11 |
| Certainty of evidence | 22 | Present assessments of certainty (or confidence) in the body of evidence for each outcome assessed. | Page 10-11 |
| **DISCUSSION** | | |  |
| Discussion | 23a | Provide a general interpretation of the results in the context of other evidence. | Page 11-12 |
|  | 23b | Discuss any limitations of the evidence included in the review. | Page 13 |
|  | 23c | Discuss any limitations of the review processes used. | Page 13 |
|  | 23d | Discuss implications of the results for practice, policy, and future research. | Page 12 |
| **OTHER INFORMATION** | | |  |
| Registration and protocol | 24a | Provide registration information for the review, including register name and registration number, or state that the review was not registered. | Page 5 |
|  | 24b | Indicate where the review protocol can be accessed, or state that a protocol was not prepared. | Page 5 |
|  | 24c | Describe and explain any amendments to information provided at registration or in the protocol. | None |
| Support | 25 | Describe sources of financial or non-financial support for the review, and the role of the funders or sponsors in the review. | Page 14 |
| Competing interests | 26 | Declare any competing interests of review authors. | Page 15 |
| Availability of data, code and other materials | 27 | Report which of the following are publicly available and where they can be found: template data collection forms; data extracted from included studies; data used for all analyses; analytic code; any other materials used in the review. | Page 14 |

**[Supplementary Table 2.](#_Toc49954750)** [CENTRAL, Clinicaltrials.gov, EMBASE, PubMed, WHO ICTRP Search strategy](#_Toc49954750)

| CENTRAL Search strategy | |
| --- | --- |
| S1 | MeSH descriptor: [Tranexamic Acid] explode all trees |
| S2 | (tranexamic acid):ti,ab,kw OR (TXA):ti,ab,kw OR (AMCHA):ti,ab,kw OR (AMCA):ti,ab,kw OR (cyklokapron):ti,ab,kw OR (Kabi 2161):ti,ab,kw OR (transamin):ti,ab,kw OR (ugurol):ti,ab,kw OR (trans-aminomethylcyclohexanecarboxylic acid):ti,ab,kw OR (spotof):ti,ab,kw OR (amchafibrin):ti,ab,kw OR (exacyl):ti,ab,kw |
| S3 | S1 OR S2 |
| S4 | MeSH descriptor: [Intracranial Hemorrhages] explode all trees OR MeSH descriptor: [Cerebral Hemorrhage] explode all trees |
| S5 | intracerebral haemorrhage OR intracranial hemorrhage OR cerebral hemorrhage OR hemorrhagic stroke OR ICH |
| S6 | S4 OR S5 |
| S7 | S3 AND S6 |
| S8 | Filter: Trials |
| Clinical Trials.gov Search strategy | |
| Advanced search, no date limit applied | |
| Condition or disease: (brain OR cerebral OR intracranial OR intracerebral OR stroke) | |
| AND | |
| Other terms: (tranexamic) | |
| EMBASE Search strategy | |
| 1 | exp tranexamic acid/ |
| 2 | 'tranexamic acid':ti,ab OR 'trans-4-(aminomethyl) cyclohexanecarboxylic acid':ti,ab OR 'TXA':ti,ab OR 'AMCHA':ti,ab OR 'AMCA':ti,ab OR 'cyklokapron':ti,ab OR 'Kabi 2161':ti,ab OR 'transamin':ti,ab OR 'ugurol':ti,ab OR 'exacyl':ti,ab |
| 3 | 1 OR 2 |
| 4 | exp basal ganglion hemorrhage/ OR exp brain hemorrhage/ |
| 5 | 'hemorrhagic stroke':ti,ab OR 'haemorrhagic stroke':ti,ab OR 'ICH':ti,ab |
| 6 | 'intracerebral':ti,ab OR 'intracranial':ti,ab OR 'cerebral':ti,ab |
| 7 | 'haematoma*':ti,ab OR 'hematoma*':ti,ab OR 'haemorrhag*':ti,ab OR 'hemorrhag*':ti,ab |
| 8 | 6 AND 7 |
| 9 | 4 OR 5 OR 8 |
| 10 | 'randomized controlled trial':de OR 'crossover procedure':de OR 'double-blind procedure':de OR 'single-blind procedure':de |
| 11 | 'random*':de,ab,ti OR 'factorial*':de,ab,ti OR 'crossover*':de,ab,ti OR 'cross NEXT/1 over*':de,ab,ti OR 'placebo*':de,ab,ti OR 'doubl* NEAR/1 blind*':de,ab,ti OR 'singl* NEAR/1 blind*':de,ab,ti OR 'assign*':de,ab,ti OR 'allocat*':de,ab,ti OR 'volunteer*':de,ab,ti |
| 12 | 10 OR 11 |
| 13 | 3 AND 9 AND 12 |
| PubMed Search strategy | |
| #1 | "Tranexamic Acid"[MeSH Terms] |
| #2 | tranexamic acid[All Fields] OR TXA[All Fields] OR AMCA[All Fields] OR AMCHA[All Fields] OR t-AMCHA[All Fields] OR anvitoff[All Fields] OR cyklokapron[All Fields] OR ugurol[All Fields] OR KABI 2161[All Fields] OR spotof[All Fields] OR transamin[All Fields] OR amchafibrin[All Fields] OR exacyl[All Fields] OR trans-4-(aminomethyl)cyclohexanecarboxylic acid[All Fields] |
| #3 | #1 OR #2 |
| #4 | "Hemorrhagic Stroke"[MeSH Terms] OR "Intracranial Hemorrhages"[MeSH Terms] |
| #5 | hemorrhagic stroke[All Fields] OR haemorrhagic stroke[All Fields] OR ICH[All Fields] |
| #6 | intracerebral[All Fields] OR intracranial[All Fields] OR cerebral[All Fields] |
| #7 | haematoma*[All Fields] OR hematoma*[All Fields] OR haemorrhag*[All Fields] OR hemorrhag*[All Fields] |
| #8 | #6 AND #7 |
| #9 | #4 OR #5 OR #8 |
| #10 | randomized controlled trial[Publication Type] OR controlled clinical trial[Publication Type] |
| #11 | "clinical trials as topic"[MeSH Major Topic] |
| #12 | randomized[Title/Abstract] OR randomised[Title/Abstract] OR randomly[Title/Abstract] OR placebo[Title/Abstract] OR trial[Title] |
| #13 | "Humans"[MeSH Terms] |
| #14 | "Animals"[MeSH Terms] |
| #15 | #10 OR #11 OR #12 |
| #16 | #13 AND #14 |
| #17 | #14 NOT #16 |
| #18 | #15 NOT #17 |
| #19 | #3 AND #9 AND #18 |
| WHO ICTRP Search strategy | |
| Condition: "brain" OR "cerebral" OR "intracranial" OR "intracerebral" (without synonyms box unchecked) | |
| AND | |
| Intervention: "tranexamic" (without synonyms box unchecked) | |
| AND | |
| Recruitment status: "all" | |
| Phases: "all" | |

**Supplementary Table 3.** Assessment of risk of bias of individual trials

Arumugam (2015)

| **Bias** | **Authors' judgement** | **Support for judgement** |
| --- | --- | --- |
| Random sequence generation  (selection bias) | Low risk | Patients or their family members randomly chose one envelope from a box containing 30 closed envelopes. Each envelope represented either the drug or control group, which was assigned using a random sequence programmer. |
| Allocation concealment  (selection bias) | Unclear risk | Not report |
| Blinding of participants and personnel  (performance bias) | Low risk | Patients in the drug group were blinded and received a rapid administration of TXA (1 g diluted in 100 mL of 0.9% saline) over a period of 10 min. |
| Blinding of outcome assessment  (detection bias) | Low risk | After 24 hours, another CT brain scan was performed, and a blinded radiologist evaluated the size and volume of the haematoma. |
| Incomplete outcome data  (attrition bias) | Low risk | Analysis for the primary outcome was complete in ITT analysis. |
| Selective reporting  (reporting bias) | Low risk | All outcomes in the paper were reported. |
| Other bias | Unclear risk | _ |

STOP-AUST

| **Bias** | **Authors' judgement** | **Support for judgement** |
| --- | --- | --- |
| Random sequence generation  (selection bias) | Low risk | Patients were randomly assigned to receive either placebo or tranexamic acid (1:1) using a centralised web-based procedure with randomly permuted blocks of varying size. |
| Allocation concealment  (selection bias) | Low risk | The investigational product was distributed to participating centres in externally indistinguishable sealed treatment kits containing either tranexamic acid or placebo. |
| Blinding of participants and personnel  (performance bias) | Low risk | Patients and all those involved in patient management orclinical or imaging assessment of adverse events or outcomes were masked to treatment allocation. |
| Blinding of outcome assessment  (detection bias) | Low risk | Patients and all those involved in patient management orclinical or imaging assessment of adverse events or outcomes were masked to treatment allocation. |
| Incomplete outcome data  (attrition bias) | Low risk | Analysis for the primary outcome was complete in ITT analysis. |
| Selective reporting  (reporting bias) | Low risk | All outcomes in the paper were reported. |
| Other bias | Unclear risk | _ |

TICH-1

| **Bias** | **Authors' judgement** | **Support for judgement** |
| --- | --- | --- |
| Random sequence generation  (selection bias) | Low risk | Computerized randomization was performed 2:1 (TA:-placebo) with minimization on age, sex, baseline severity (National Institutes of Health Stroke Scale [NIHSS]), and time from stroke onset. |
| Allocation concealment  (selection bias) | Low risk | All image analyses were performed blinded to  clinical status and treatment allocation. |
| Blinding of participants and personnel  (performance bias) | Unclear risk | Not report |
| Blinding of outcome assessment  (detection bias) | Low risk | All image analyses were performed blinded to clinical status and treatment allocation. |
| Incomplete outcome data  (attrition bias) | Low risk | No patients were lost to follow-up |
| Selective reporting  (reporting bias) | Low risk | All outcomes in the paper were reported. |
| Other bias | Unclear risk | _ |

TICH-2

| **Bias** | **Authors' judgement** | **Support for judgement** |
| --- | --- | --- |
| Random sequence generation  (selection bias) | Low risk | A secure website was used to randomly assign all participants eligible for inclusion to receive tranexamic acid or matching placebo, with 1:1 allocation. |
| Allocation concealment  (selection bias) | Low risk | The random allocation sequence was generated by the trial programmer. Sharp Clinical Services (Crickhowell, UK) prepared individual masked treatment packs containing four 5 mL glass ampoules of tranexamic acid 500 mg or sodium chloride 0·9%, which were made identical in appearance bythe addition of a heat shrink sleeve. Ampoules and the treatment pack were labelled with a unique pack number. Sharp Clinical Services stored the treatment packs and distributed them to pharmacies within trial sites using a web-based system of control. |
| Blinding of participants and personnel  (performance bias) | Low risk | Treatment allocation was concealed from all staff and patients involved in the trial. |
| Blinding of outcome assessment  (detection bias) | Low risk | Treatment allocation was concealed from all staff and patients involved in the trial. |
| Incomplete outcome data  (attrition bias) | Low risk | The primary outcome of mRS at day 90 was assessed in 2307 (99%) of 2325 participants |
| Selective reporting  (reporting bias) | Low risk | All outcomes in the paper were reported |
| Other bias | Unclear risk | _ |

TRAIGE

| **Bias** | **Authors' judgement** | **Support for judgement** |
| --- | --- | --- |
| Random sequence generation  (selection bias) | Low risk | Patients were randomly assigned to receive either placebo (0.9% NaCl) or tranexamic acid (1:1) using a computer-generated procedure with randomly permuted blocks of varying size. |
| Allocation concealment  (selection bias) | Low risk | The investigational product was distributed to participating centers in externally indistinguishable sealed treatment kits containing either tranexamic acid or placebo in identical standard off-the-shelf ampoules. |
| Blinding of participants and personnel  (performance bias) | Low risk | Treatment allocation was concealed from all patients and investors involved in the trial. |
| Blinding of outcome assessment  (detection bias) | Low risk | Treatment allocation was concealed from all patients and investors involved in the trial. |
| Incomplete outcome data  (attrition bias) | Low risk | Analysis for the primary outcome was complete in ITT analysis. |
| Selective reporting  (reporting bias) | Low risk | All outcomes in the paper were reported. |
| Other bias | Unclear risk | _ |

**[Supplementary Table 4.](#_Toc49954757)** [GRADE assessment on the certainty of evidence](#_Toc49954757)

| **Outcome** | **Trials, n** | **Study design** | **Risk of bias** | **Inconsistency** | **Indirectness** | **Imprecision** | **Other considerations** | **Certainty of evidence** |
| --- | --- | --- | --- | --- | --- | --- | --- | --- |
| HE | 5 | Randomized | Not serious | Not serious | Not serious | Serious^*^ | None | Moderate |
| PFO  (3 mo) | 4 | Randomized | Not serious | Not serious | Not serious | Not serious | None | High |
| Mortality (3 mo) | 4 | Randomized | Not serious | Not serious | Not serious | Not serious | None | High |
| MTE | 4 | Randomized | Not serious | Not serious | Not serious | Serious^*^ | None | Moderate |

^*^Wide confdence intervals do not exclude important beneft or harm which lowers our certainty in efect

**Supplement Figure 1.** Funnel plot and Egger's test. (A) hematoma expansion, (B) 3-month poor functional outcome, (C) 3-month mortality, and (D) major thromboembolic events

(A)


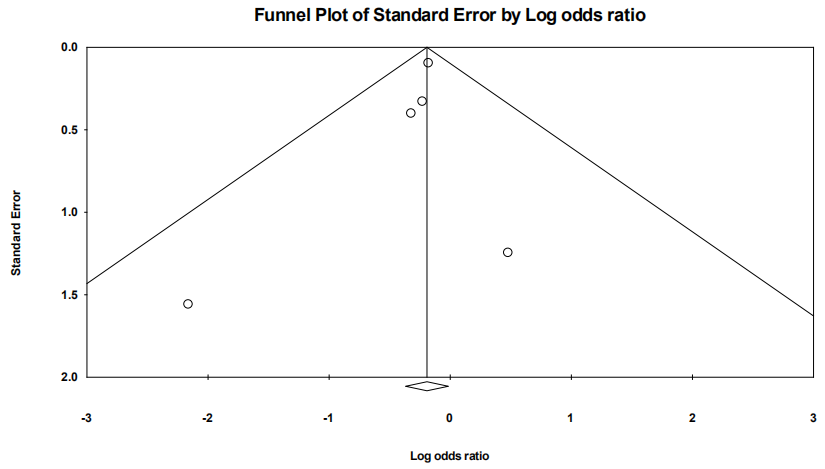


Egger's test for publication bias: *p* = 0.830.

(B)


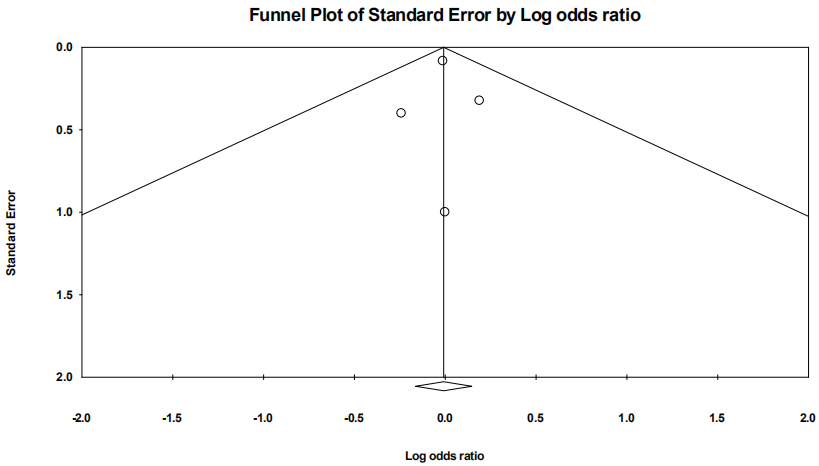


Egger's test for publication bias: *p* = 0.996.

(C)


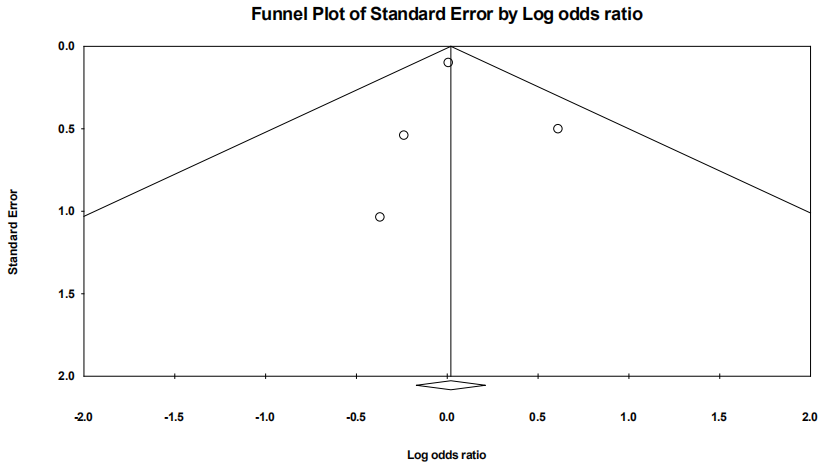


Egger's test for publication bias: *p* = 0.882.

(D)


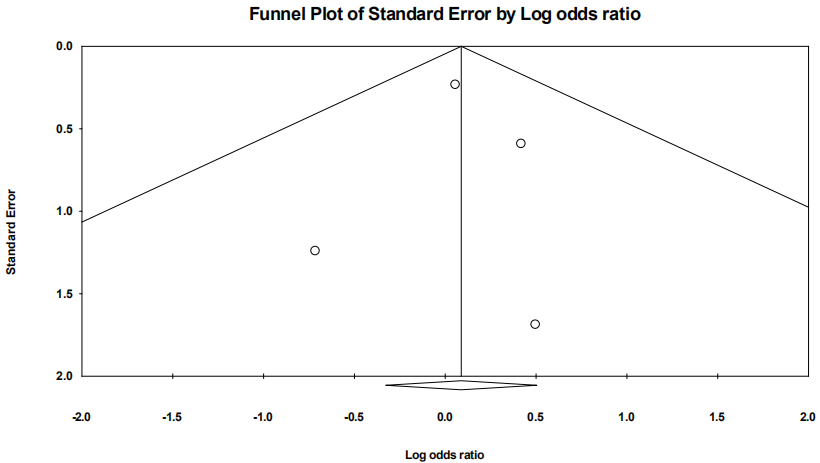


Egger's test for publication bias: *p* = 0.863.

**Supplementary Appendix 1.** Post-hoc sensitivity analysis

| **Outcome** | **Trials, n** | **Result** | | |
| --- | --- | --- | --- | --- |
|  |  | **OR (95% CI)** | ***p* Value** | **Heterogeneity**  **(I^2^, *p* for Cochran Q)** |
| HE | 3 | 0.828 (0.693-0.988) | 0.036 | I^2^ = 0.000%, *p =* 0.935 |
| PFO (3 mo) | 3 | 0.991 (0.849-1.158) | 0.913 | I^2^ = 0.000%, p = 0.701 |
| Mortality (3 mo) | 3 | 1.024 (0.845-1.239) | 0.812 | I^2^ = 0.000%, *p =* 0.444 |
| MTE | 3 | 1.085 (0.713-1.650) | 0.703 | I^2^ = 0.000%, *p =* 0.688 |

Abbreviations: CI = confidence interval; HE = hematoma expansion; MTE = major thromboembolic events; OR = odd ratio; PFO = poor functional outcome.

HE


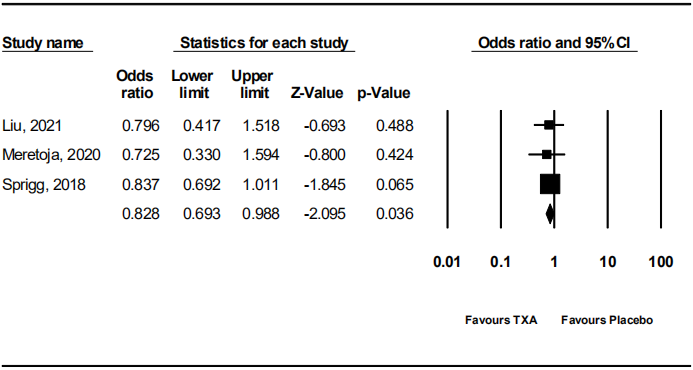


PFO (3 mo)


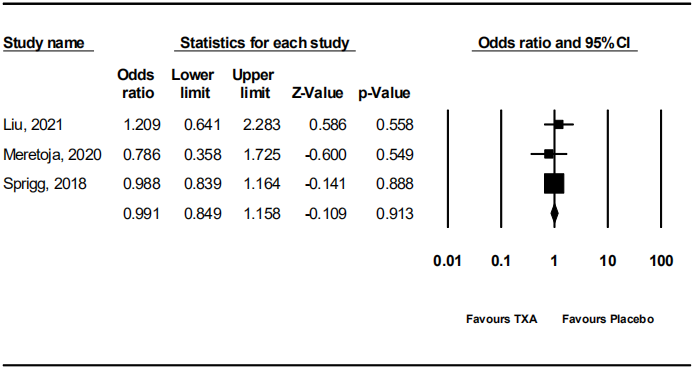


Mortality (3 mo)


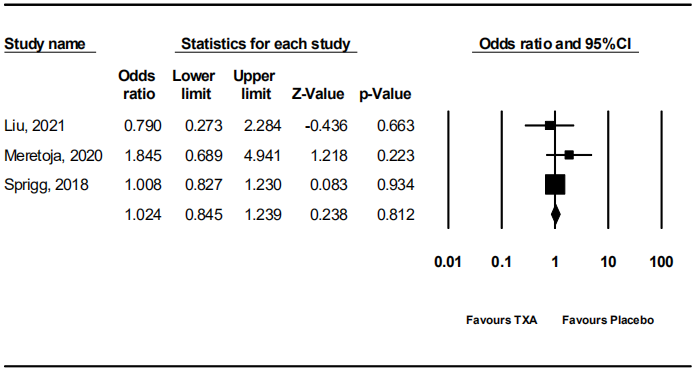


MTE


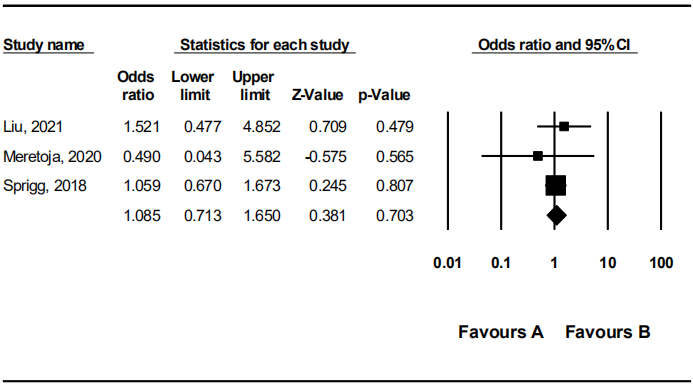


**Supplementary Appendix 2.** Subgroup analysis

| **Outcome** | **Result (****standard-risk population)** | | | **Result (high-risk population)** | | |
| --- | --- | --- | --- | --- | --- | --- |
|  | **OR**  **(95% CI)** | ***p* Value** | **Heterogeneity**  **(I^2^, *p* for Cochran Q)** | **OR**  **(95% CI)** | ***p* Value** | **Heterogeneity**  **(I^2^, *p* for Cochran Q)** |
| HE | 0.834  (0.690-1.007) | 0.059 | I^2^ = 0.000%, *p =* 0.388 | 0.646  (0.503-0.829) | 0.001 | I^2^ = 0.000%, *p =* 0.886 |
| PFO  (3 mo) | 0.988  (0.839-1.164) | 0.889 | I^2^ = 0.000%, *p* = 0.991 | 0.844  (0.633-1.127) | 0.251 | I^2^ = 0.000%, p = 0.739 |
| Mortality (3 mo) | 1.005  (0.825-1.224) | 0.961 | I^2^ = 0.000%, *p =* 0.718 | 1.350  (0.743-2.454) | 0.325 | I^2^ = 0.000%, *p =* 0.498 |
| MTE | 1.068  (0.679-1.679) | 0.777 | I^2^ = 0.000%, *p =* 0.796 | 1.209  (0.497-2.940) | 0.676 | I^2^ = 0.000%, *p =* 0.710 |

Abbreviations: CI = confidence interval; HE = hematoma expansion; MTE = major thromboembolic events; OR = odd ratio; PFO = poor functional outcome.

PFO (3 mo): (A) standard-risk population and (B) high-risk population


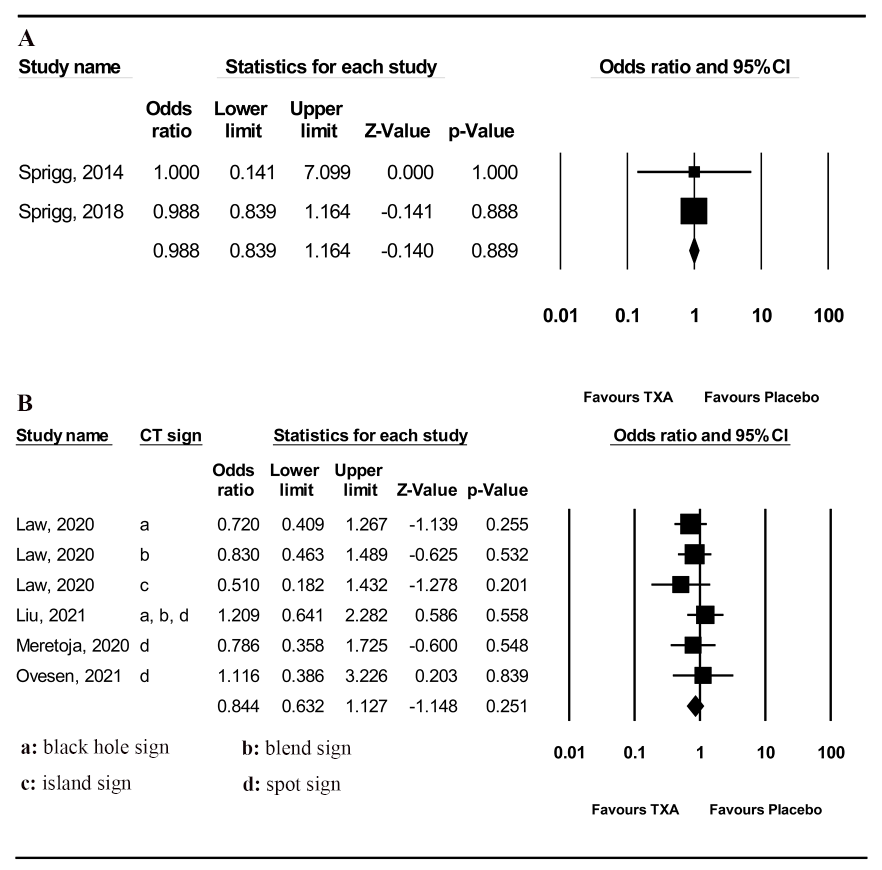


Mortality (3 mo): (A) standard-risk population and (B) high-risk population


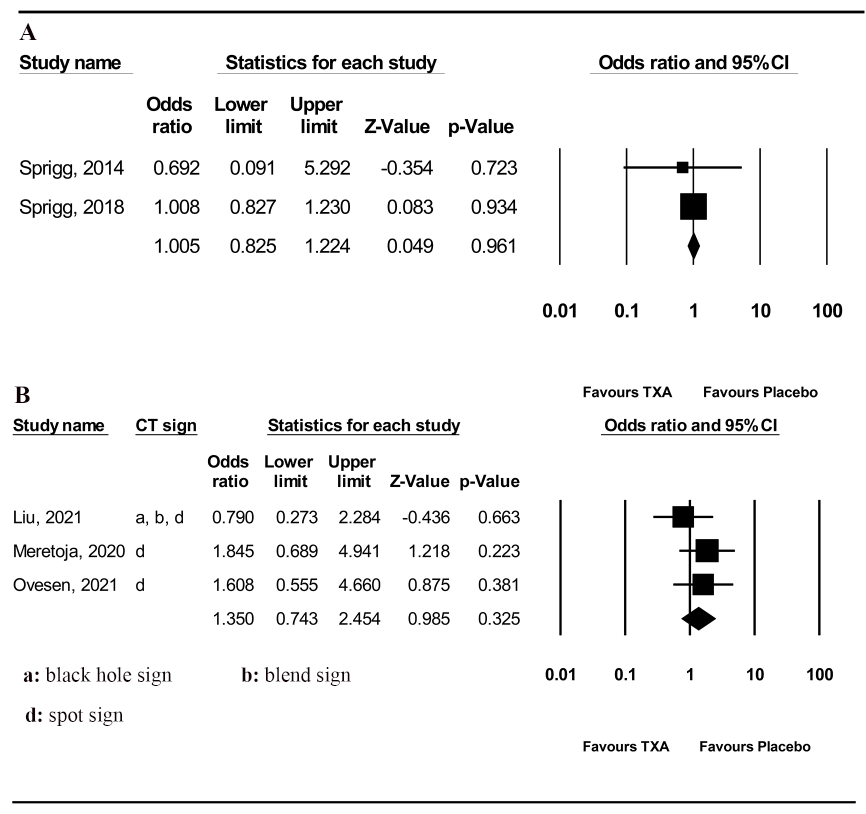


MTE: (A) standard-risk population and (B) high-risk population


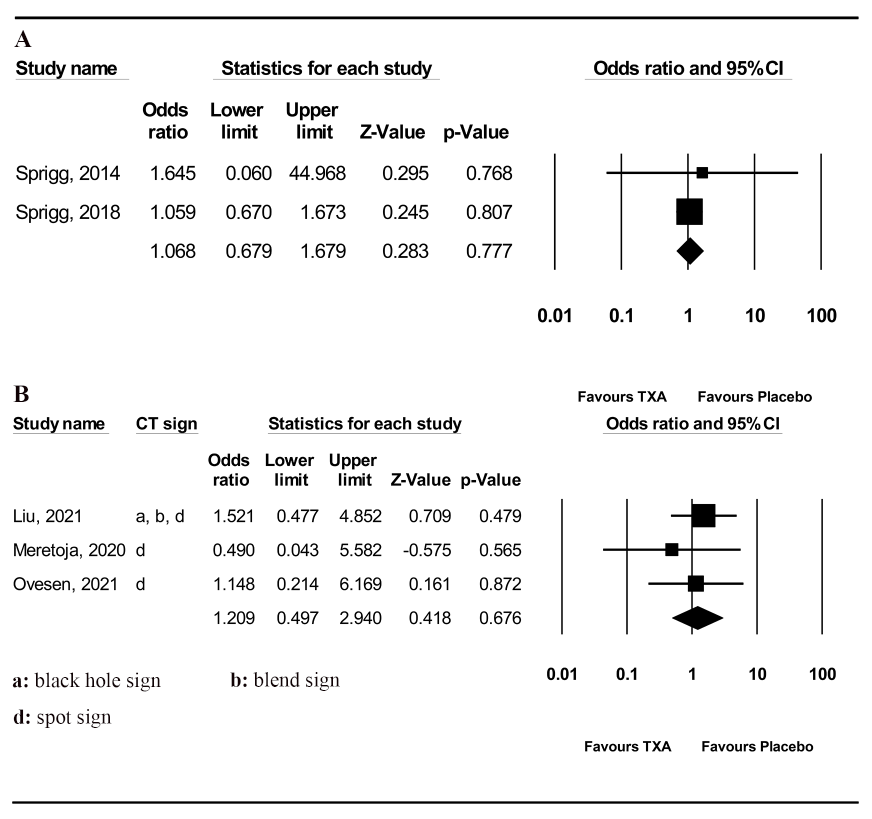

Supplement: Supplementary file 1 [file Data_Sheet_1.docx]
